# Correlation analysis of the total IgY level in hen serum, egg yolk and offspring serum

**DOI:** 10.1186/2049-1891-4-10

**Published:** 2013-03-08

**Authors:** Hancong Sun, Sirui Chen, Xia Cai, Guiyun Xu, Lujiang Qu

**Affiliations:** 1 Department of Animal Genetics and Breeding, National Engineering Laboratory for Animal Breeding, College of Animal Science and Technology, China Agricultural University, Beijing 100193, China

**Keywords:** Chicken, Correlation, Indirect ELISA, Total IgY

## Abstract

The correlation between IgY levels of the serum and the yolk has been well documented in wild and domestic birds. The levels of total yolk IgY can be an index of the general health status of birds and may contribute to breeding programs when fitness of the offspring is a concern. We measured the levels of total serum IgY and yolk IgY in three different breeds (White Leghorn, Silkie and Dongxiang blue-shell) using indirect ELISA, and found that there was a significantly positive correlation between the levels of total serum IgY and total yolk IgY in all three breeds (White Leghorn: r = 0.404, *P* < 0.001, n = 100; Silkie: r = 0.561, *P* < 0.001, n = 70; Dongxiang blue-shell: r = 0.619, *P* < 0.001, n = 30). We also measured the total serum IgY levels in the 3-day-old offspring hatched from the Silkie hens and results were significantly correlated for serum IgY levels (r = 0.535, *P* < 0.001, n = 70) and the yolk IgY levels (r = 0.481, *P* < 0.001, n = 70). The regression analysis showed simple linear regression between IgY levels in hen serum, yolk and offspring serum. Our results suggest that total IgY level could be used as an index for chicken fitness.

## Background

The mechanisms of antibody transmission from hen to yolk and the use of antibodies by offspring in chickens are well documented [1-6]. In chickens, as in mammals, IgG (IgY) is the predominant antibody isotype transferred from hen to offspring via the eggs [2,7]. Chicken IgY is first transported from the serum of the hen to the yolk by a selective IgY transport system in avian ovarian follicles that recognizes the Fc portion of IgY and excludes polymeric immunoglobulins [8]. It has been reported that the uptake of IgY in yolk is proportional to yolk mass accumulation, and so the concentration of IgY is invariant [3]. The yolk IgY is also absorbed into fetal circulation during incubation. The circulating IgY in young is principally of endogenous origin within 2 wk [9] and offspring begin to actively synthesize IgY from 5 d post-hatch [7,10]. Maternally derived antibodies (IgY) provide the first form of humoral immunity for offspring early in life and improve offspring performance and survival [11].

Because of its low cost, high production efficiency and good biochemical properties, IgY is widely used in immunodiagnostics and immunotherapy [12]. Significant growth of IgY literature shows an interest in antibody production from chickens and the extraction of antigen-specific antibody from egg yolk [13-15]. To date, chicken IgY and its application are still intriguing.

The concentration of total serum IgY in the chicken can be indicative of the fitness, health and nutritional state of the birds [11]. Here, we measured the concentration of total IgY of chickens and analyzed the correlation among the total IgY levels in hen serum and yolk, and offspring serum in three chicken breeds. The results provide an important index for selection of IgY levels in hen serum when the fitness of newly hatched chicks is a concern. This will contribute to the selection of better hens for antigen-specific IgY production by measuring maternal serum IgY levels.

## Methods

### Sampling

A total of 70 44-week-old Silkie hens, 30 44-week-old Dongxiang blue-shelled layer hens and 100 44-week-old White Leghorn hens were used in this study. For each breed, the hens were kept in the same conditions and were vaccinated similarly. First, 2 mL blood was collected via the wing vein and three eggs from each hen were collected 1 weeks after bleeding. The blood samples were stored at 4°C for 1 h and centrifuged at 3,000 × *g* for 10 min at 4°C. The liquid that remained after blood had clotted was collected and stored as serum. Yolk IgY was isolated using the method reported by Müller *et al*. [16]. Briefly, the yolk samples were diluted 1:1 in distilled water, and centrifuged at 10,000 × *g* for 25 min. The supernatants were collected and the pH was adjusted to pH 5.0 ~ 5.2 using 1 mol/L HCl to reach reaction condition. All the serum and yolk IgY samples were kept at -20°C.

To analyze the correlation between serum IgY levels of the hens and their offspring, we collected three consecutively laid fertile eggs from Silkie hens and hatched the offspring shortly after for yolk IgY level measurement. All the eggs were hatched together. Next, 1 mL of blood was collected via the jugular vein from 3-day-old chickens and the serum was extracted as described above.

### Measurement of total IgY

Total IgY in both serum and yolk samples were measured using a sandwich ELISA protocol [16] with minor modifications. Shortly, NuncTM 96-well ELISA plates were coated with rabbit anti-chicken IgY antibody (5 mg/mL; 1:10,000 diluted in PBS, United States Biological, CA, USA) by overnight incubation at 4°C. After emptying the wells, the plates were washed with 0.05% PBS-Tween three times (1 min/time), incubated with 1.5% BSA-PBS for 1 h at 37°C, and again washed with 0.05% PBS-Tween three times (1 min/time). Fifty microliters of each sample was added in triplicate to the wells. We also used dilution solution as a negative control and chicken IgY (0.2 μg/mL, Promega, USA) as a positive control. The plates were incubated for 3 h at 37°C, and then washed with 0.05% PBS-Tween (three times). Subsequently, an alkaline phosphatase conjugated goat anti-chicken IgY (United States Biological, 1 mg/mL, 1:5000 diluted in 1.5% BSA-PBS) was added. Plates were incubated for another night at 4°C. In the last step, plates were washed with 0.05% PBS-Tween (three times) and 80 μl of an alkaline phosphatase substrate (PNPP, p-nitro phenol phosphate, Sigma 104 phosphatase substrate in diethanol amine buffer (1 mg/mL); Sigma, USA) was applied and kept in a dark place. The absorbance (OD value) was measured at 405 nm in an ELISA reader (Thermo Labsystem MK3, Thermo, USA) 45 min after adding the substrate.

Prior to ELISA analysis, the working solutions of yolk and serum samples were predetermined for all three breeds. For this, we prepared a pool of all yolk and all serum samples as standard solutions, which were diluted into a ten-fold series. The pooled ten-fold series samples were measured by sandwich ELISA to form a standard curve (data not shown). The working dilution was determined by the middle point of the linear part of the standard curve. After the working solutions were determined for each breed, all the samples were diluted by the working dilution, and measured using the sandwich ELISA method.

### Data analysis

We used the SPSS10.0 statistical software package to perform Pearson correlation coefficient (two-tail) and regression analysis. The correlations of the serum and yolk IgY levels of the hens were analyzed in the three breeds. For the Silkie breed, the IgY levels of the offspring serum, hen serum and the yolk were also analyzed for correlation and regression.

## Results

The working dilutions of the samples are shown in Table 1. The average absorbance value of OD_405_ of the three replications was used as the IgY level.

**Table 1:** Working dilutions of the samples

**Samples**	**Working dilution**^1^
Dongxiang blue-shelled yolk	1:1.5 × 10^5^
Dongxiang blue-shelled serum	1:10^5^
White Leghorn yolk	1:5.5 × 10^5^
White Leghorn serum	1:5.5 × 10^5^
Silkie yolk	1:10^5^
Silkie hen serum	1:10^5^
Silkie offspring serum	1:10^5^

^1^The samples were diluted with 1.5% BSA-PBS into working dilution.

**Figure 1:**
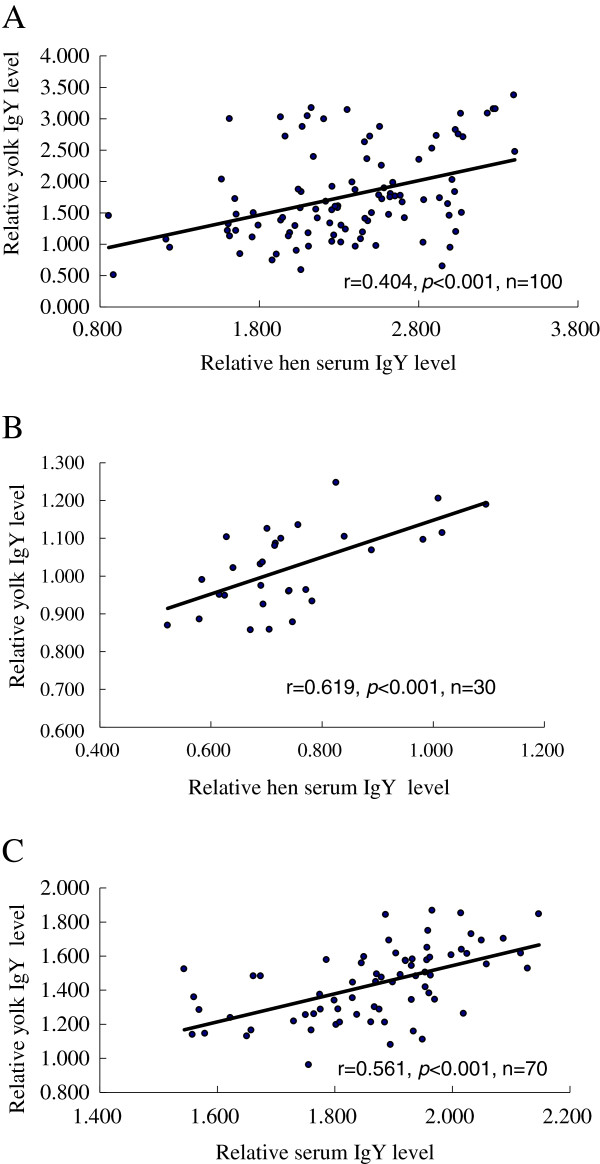
**Correlation between the serum and egg yolk total IgY levels in Dongxiang (A), White Leghorn (B) and Silkie (C) chickens. **The relative IgY levels are presented as the absorbance units (OD values). The r is the correlation coefficient and n is the number of samples.

We found there was a significant correlation between the hen serum IgY and yolk IgY in the three breeds. The correlation of Dongxiang (r = 0.619, *P <* 0.001, n = 30, Figure 1A) was the highest among the three breeds (White Leghorn: r = 0.404, *P* < 0.001, n = 100, Figure 1B; Silkie: r = 0.561 *P* < 0.001, n = 70, Figure 1C).

**Figure 2:**
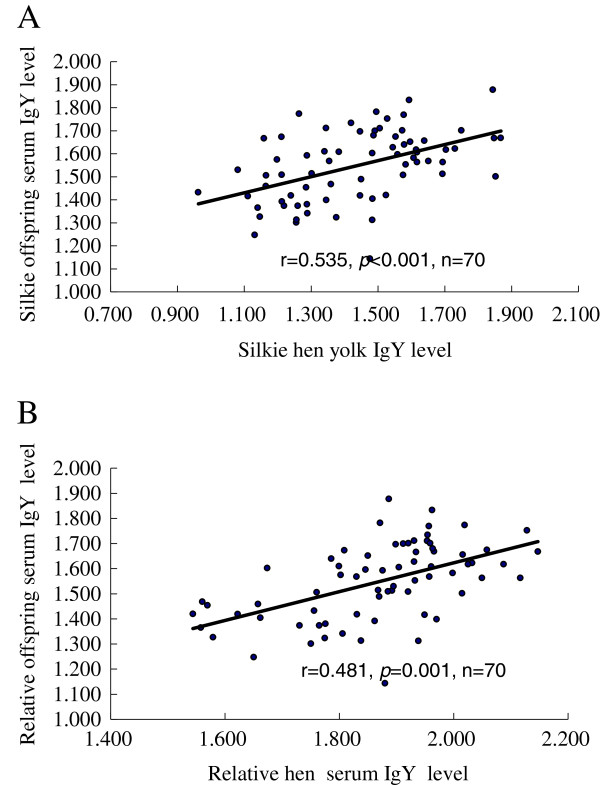
**Correlation of Silkie offspring serum IgY levels with Silkie hen yolk IgY (A), and Silkie hen serum IgY levels (B). **The relative IgY levels are presented as the absorbance units (OD values). The r is the correlation coefficient and n is the number of samples.

We also found the same tendency among hen serum IgY, egg yolk IgY and offspring serum IgY. The IgY level in offspring serum was significantly correlated with hen serum IgY (r = 0.535, *P* = 0.001, n = 70, Figure 2A) and with egg yolk IgY (r = 0.481, *P* = 0.001, n = 70, Figure 2B).

The regression equation was made for each breed as follows: y_1_ = 0.489x_1_ + 0.659, *P*_1_ < 0.001, (y_1_: OD_405_ of Dongxiang yolk IgY, x_1_: OD_405_ of Dongxiang serum IgY); y_2_ = 0.549x_2_+ 0.479, *P*_2_ < 0.05, (y_2_: OD_405_ of White Leghorn yolk IgY, *x*_2_: OD_405_ of White Leghorn serum IgY); y_3_ = 0.827x_3_ – 0.109, *P*_3_ < 0.001, (y_3_: OD_405_ of Silkie yolk IgY, x_3_: OD_405_ of Silkie hen serum IgY); y_4_ = 0.350x_4_ + 1.046, *P*_4_ < 0.001, (y_4_: OD_405_ of Silkie offspring serum IgY, x_4_: OD_405_ of Silkie yolk IgY); y_5_ = 0.574x_5_ + 0.476, *P*_5_ < 0.05 (y_5_: OD_405_ of Silkie offspring serum IgY, x_5_: OD_405_ of Silkie hen serum IgY).

## Discussion

The transfer of passive immunity from mother to young has long been known to occur in birds as well as mammals [7]. IgY, the avian and reptilian counterpart of mammalian IgG, is taken to egg yolk from blood and transported across yolk sac membranes during development [3,17]. Here, we found that there was a significant correlation between the level of total serum IgY and total yolk IgY in White Leghorn, Silkie and Dongxiang blue-shell chickens. Hen IgY levels were positively associated with the egg yolk IgY levels. This indicates that hens with higher IgY levels may in turn lay eggs with higher IgY levels, which suggests that hens of good immune state were able to allocate more immune defense ability to eggs and their offspring. In birds, maternal IgY in egg yolk is transferred across the yolk sac to passively immunize offspring during gestation and early independent life. The transmission of high levels of passive immunity via the mother has been shown to enhance disease resistance of chickens. Our results concur with those of a previous study where the total IgY levels in hens, yolk and offspring were also strongly correlated in broiler breeder lines [2].

## Conclusions

We found a significant correlation among IgY levels in hen serum, yolk and offspring serum in White Leghorn, Silkie and Dongxiang blue-shelled chickens. We have provided a better understanding of the correction of IgY levels among hen serum, yolk and offspring serum. IgY products are widely used nowadays, especially in immunoassays. The total serum IgY concentration could also be used as an index for offspring fitness because of the positive correlation between total IgY concentration and chicken health [11].

## Abbreviations

Yolk IgY: Immunoglobulin yolk; Serum IgY: Immunoglobulin G; ELISA: Enzyme-linked immunosorbent assay; PBS: Phosphate buffered saline; BSA: Bovine serum albumin; PNPP: Disodium 4-nitrophenyl phosphate.

## Competing interests

The authors declare that they have no competing interests.

## Authors’ contributions

The authors’ responsibilities were as follows: LQ designed the study; HS and XC carried out the experiments, participated in the data collection, data analysis, and drafted the manuscript; LQ, GX and SC provided technical expertise and revised the manuscript. All authors have approved the final manuscript.
